# Supplementary material for: Complement in Human Pre-implantation Embryos: Attack and Defense
Source: Front Immunol. 2019 Sep 18;10:2234. doi: 10.3389/fimmu.2019.02234 (PMC6759579; doi:10.3389/fimmu.2019.02234)
Supplement: Supplementary file 1 [file Table_1.doc]

**Complement in human pre-implantation embryos: attack and defense**

**Supplementary Tables**

| **Partially degraded transcripts / potential novel isoforms** | | | |
| --- | --- | --- | --- |
| **Oocyte** | **Zygote** | **4-cell** | **8-cell** |
| Adiponectin | Adiponectin | Adiponectin | Adiponectin |
| C1q-C | C1q-C | C1q-C | C1q-C |
| C1R | C1R | C1R | C5 |
| C7 | C3 | C7 | C7 |
| Cadherin13 | C7 | Cadherin13 | CD46 |
| CR1 | Cadherin13 | CD46 | CR1 |
| Properdin | CR1 | CR1 | Factor D |
|  | Factor B | Properdin | Properdin |
|  | Properdin |  |  |

**Supplementary Table 1: Complement gene-transcripts mapped to coding regions outside the 5’ UTR.** Highlighted here are additional complement-related transcripts mapped to coding regions outside the 5’ UTR. Based on the time-dependent regulation of gene transcription surrounding the human genome activation, it is not possible to determine the specific expression of these genes at the various stages. The massive presence of certain genes through all tested stages does however indicate that gene transcription from particular loci is active during the investigated early embryonic stage. C1q-C: C1q, C-chain, CR1: complement receptor 1.

| Complement-related genes, with no detected transcription: | C1QA, C1QB, C4A, C4B, MBL, MASP1, FCN1, FCN2, FCN3, PTX3, C6, C8A, C8B, C9, C1NH, C4BPA, CFH, CFHR1, CFHR2, CFHR3, CFHR4, CFHR5, CRIG, VTN, C5AR2, CD93, SELL, MMP8, MMP9, DMBT1 |
| --- | --- |

**Supplementary Table 2: Complement genes with no expression.** Listed are complement related genes not found to be expressed. MBL: Mannose binding lectin, MASP: MBL associated serine protease. FCN: Ficolin, PTX: Pentraxin, C1INH: C1 inhibitor, CFH: Complement factor H, CFHR: Complement factor H related, CRIG: Complement Receptor of the Immunoglobulin superfamily, VTN: Vitronectin, VSIG4, C5AR2: C5a Receptor 2, SELL: L-selectin MMP: matrix metalloproteinase, DMBT1: deleted in malignant brain tumor 1 (also known as SALSA or gp340).

**Supplementary Figures**

**Figure S1: Negative controls of confocal imaging.** Embryos were incubated directly with secondary antibodies (all from Thermo Fisher Scientific; 1:500), without the presence of primary antibodies. Images were exposed at comparable levels to the images in Figures 2-4, and overlaid with DAPI (blue). **(A)** Donkey anti-mouse AlexaFluor®594. **(B)** Donkey anti-goat AlexaFluor®488. **(C)** Donkey anti-rabbit AlexaFluor®488.

**Figure S2: Localization of CD59 from zygote through to 8-cell stage.** To support the observation of CD59 expression at all developmental stages, we stained embryos at zygote through to 8-cell stage for this marker. At the zygote stage (**A**) CD59 stains evenly on the membrane surface. As the embryo develops through 2-cell stage (**B**), 4-cell stage (**C**), and 8-cell stage (**D**), the staining gathers into particular clusters concentrated at the cellular junctions (**B**-**D**). Displayed are 2PN embryos (3 PN for the zygote). Left panels: single planes, overlay of protein stain (green) and DAPI (blue). Right panels top to bottom: DAPI (blue), protein stain (green), and BF. Scale bars: 50 μm. Due to the very limited material, these stainings were only performed with n = 1 2PN embryos per stage. Zygote, plus additional 4 3PN embryos.

**Supplementary videos**

**Video 1:** Human cleavage stage IVF embryos were thawed in Vitrolife G-TL serum-free media. The embryos were incubated with anti-complement antibodies and analyzed by confocal microscopy. The analysis revealed a clear staining for CD55, particularly at cellular junctions. 3D rendering, overlay of CD55 (green) and DAPI (blue). Scale bar as indicated.

**Video 2:** Human cleavage stage IVF embryos were thawed in Vitrolife G-TL serum-free media. The embryos were incubated with anti-complement antibodies and analyzed by confocal microscopy. The analysis revealed a clear surface staining for CD59, with increased signal at cellular junctions. 3D rendering, overlay of CD59 (green) and DAPI (blue). Scale bar as indicated.
