# Supplementary figures and images for: Complement in Human Pre-implantation Embryos: Attack and Defense
Source: Front Immunol. 2019 Sep 18;10:2234. doi: 10.3389/fimmu.2019.02234 (PMC6759579; doi:10.3389/fimmu.2019.02234)

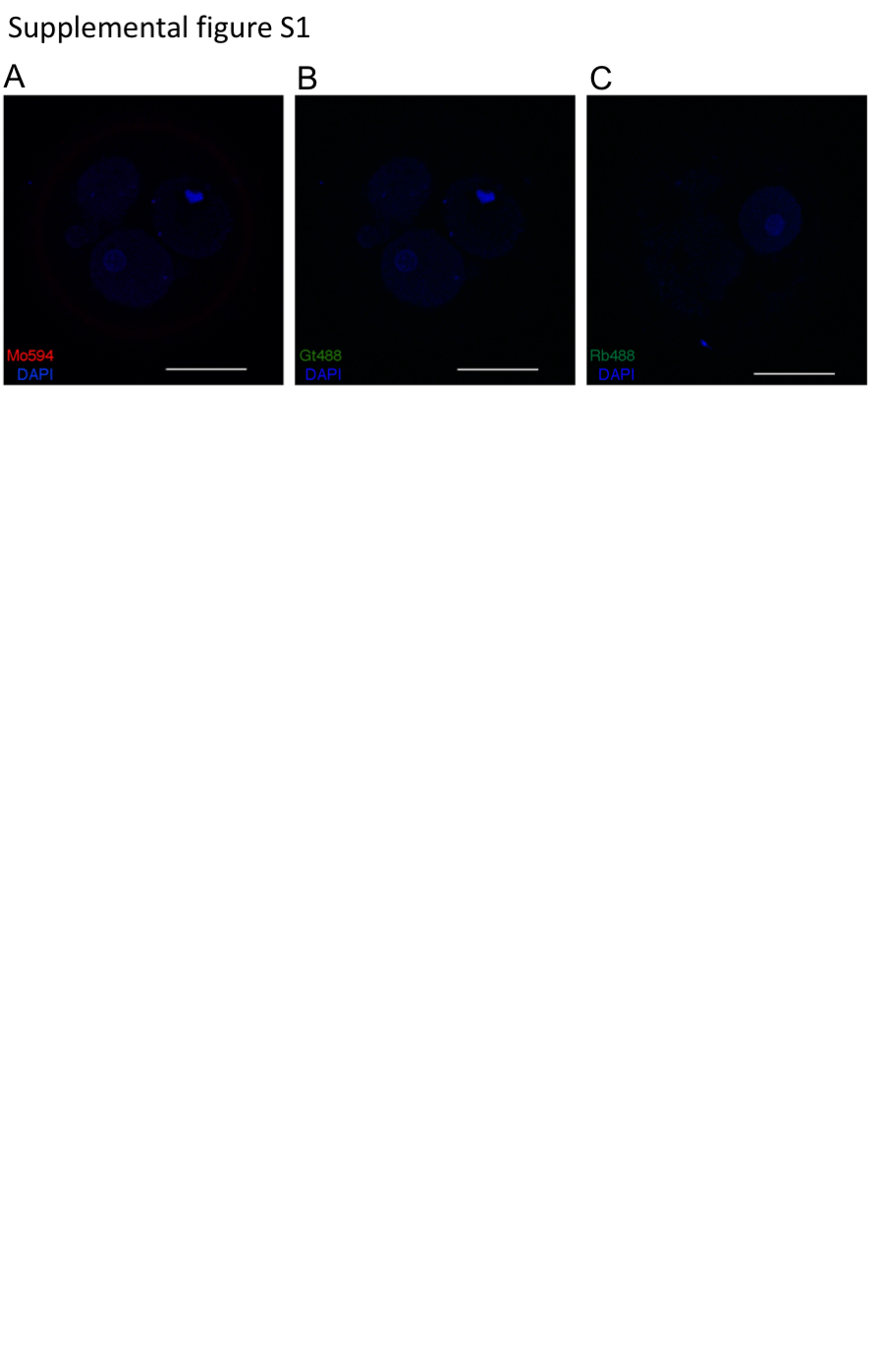

Supplement: Supplementary file 4 [file Image_1.TIFF]

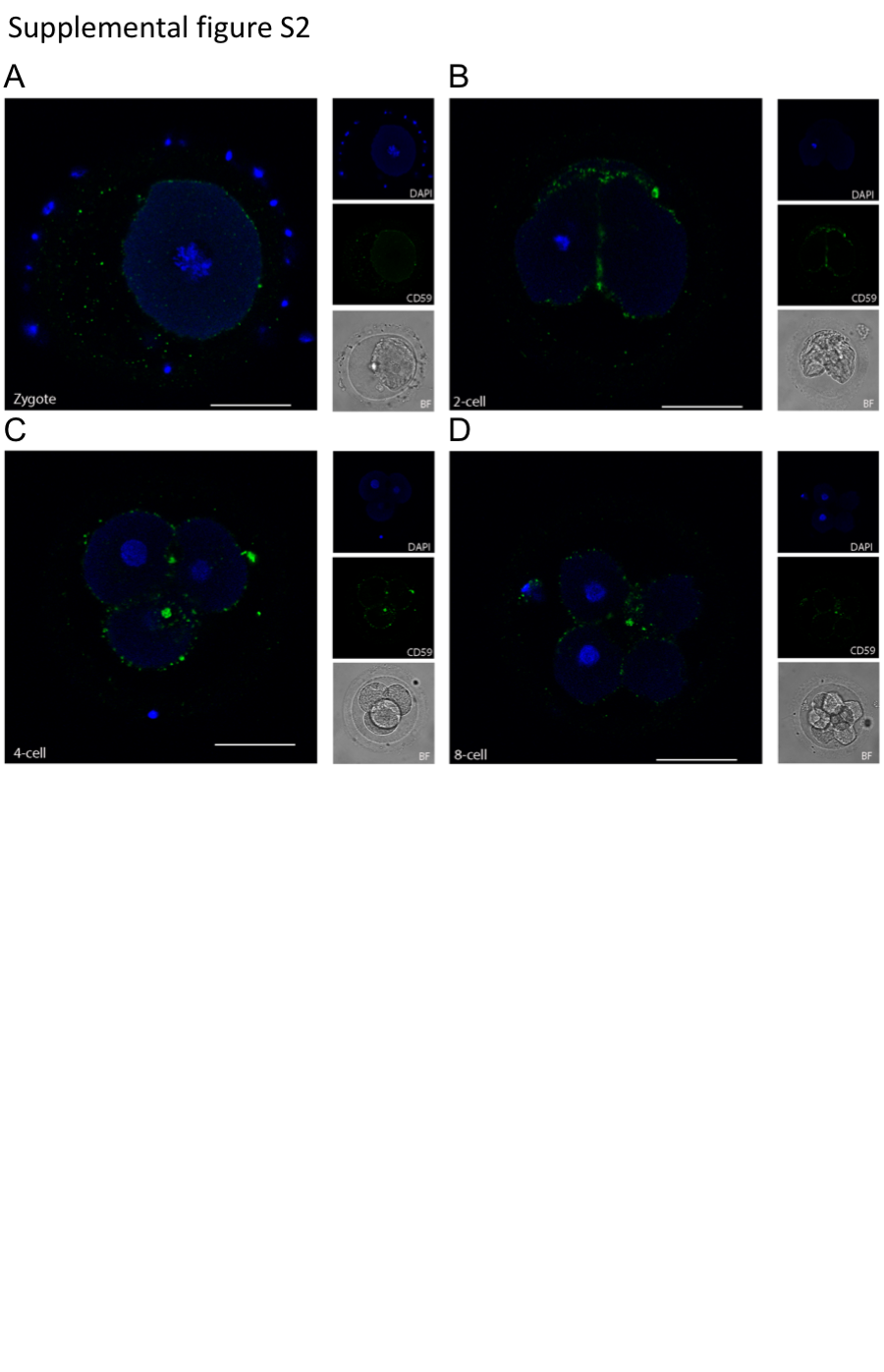

Supplement: Supplementary file 5 [file Image_2.TIFF]
